# Cavernous hemangioma of the bladder: an additional case managed by partial cystectomy and augmentation cystoplasty

**DOI:** 10.11604/pamj.2015.22.131.7838

**Published:** 2015-10-13

**Authors:** Mounir Lahyani, Amine Slaoui, Nabil Jakhlal, Tarik Karmouni, Khalid Elkhader, Abdellatif Koutani, Ahmed Ibn Attya Andaloussi

**Affiliations:** 1 Mounir Lahyani, Department of Urology B, Ibn Sina Hospital, Morocco

**Keywords:** Hemangioma, bladder, partial cystectomy, diagnosis, treatment

## Abstract

Cavernous Hemangioma of the Bladder (CHB) is benign and rare lesions. Clinical presentation has no pathognomonic signs although gross painless hematuria is the most frequent complain. CHB is suspected by cystoscopy and radiologic findings and confirmed by pathologic examinations. Management is controversial due to the bleeding risk of this highly vascularized lesion. Partial cystectomy is the treatment of choice for surgically accessible lesions. However, it appears that small lesions could be treated using transurethral resection. Since CHB is a rare case, we report another case treated successfully with a partial cystectomy associated with an augmentation cystoplasy.

## Introduction

CHB is an uncommon benign tumor that accounts for 0.6% of bladder tumors [1]. It most likely is congenital in origin, arising from embryonic angioblastic stem cells [2]. BCH is a rare cause of hematuria. It may appear in every part of the urinary tract and presents mostly in childhood. The treatment differs from partial cystectomy and endoscopic removal or laser-therapy.

## Patient and observation

**Figure 1:**
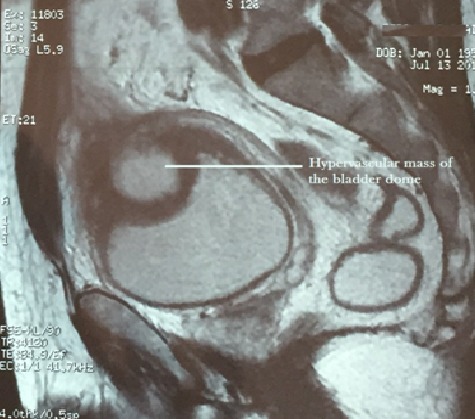
Sagittal section in MRI T2 showing the CHB

**Figure 2:**
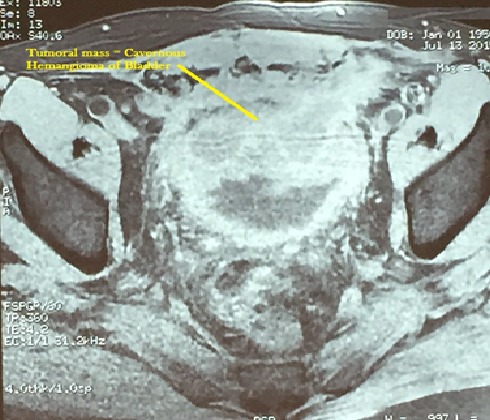
Transverse section in MRI of the CHB: T1 gadolinium injection

**Figure 3:**
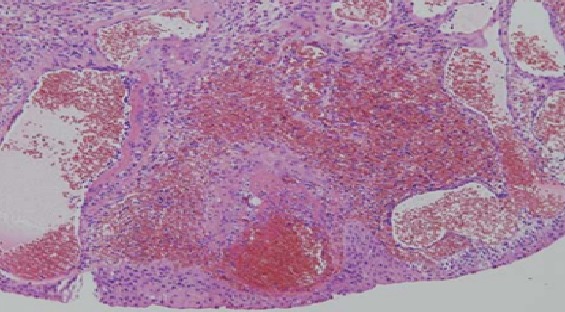
Pathology result after partial cystectomy: large and dilated vessels which are engorged with blood and covered with a thin wall

We report the case of a 60 years old patient without significant medical history, who presented a macroscopic haematuria. The etiology was a cavernous hemangioma of the bladder confirmed by histological examination of transurethral endoscopic resection chips. Two years later, irritative lower urinary tract symptoms led to the realization of an ultrasound that reveals an echogen mass in the dome of the bladder. The same hypervascular mass was indicated at the same side in abdominal pelvic MRI. It contains fluid central areas that can correspond either to necrosis or vascular ectasia. There is an extra-bladder invasion but no ganglions. The upper urinary tract was normal (Figure 1, and Figure 2). Bimanual examinations were performed. There was no adhesion to the pelvic wall. An open supra-trigonal partial cystectomy with a safe margin was performed by an intraperitoneal approach associated with an augmentation cystoplasty. The postoperative follow-up was favorable. Histopathological study confirmed the angiomatous nature of the tumoral mass (Figure 3).

## Discussion

Bladder hemangioma is a rare cause of hematuria. Although cavernous hemangioma is mostly developed in derma and epidermis, it is rarely reported in the mucosal parts. A review of the literature in 1942 revealed 40 cases of CHB had been reported up to that time [3]. Generally, there is a congenital cause for CHB. It occurs in all age groups and usually is observed in patient's age ≤ 30 years, with a slight male predominance. The most common symptom is macroscopic hematuria; other symptoms include irritative voiding symptoms and abdominal pain. It may coexist with cutaneous hemangioma or be associated with the Sturge-Weber syndrome or the Klippel-TrenaunayWeber syndrome [4–6]. The majority of hemangiomas are solitary, small (≤3 cm), and cavernous, with a predilection for the dome, posterior wall, and trigone of the bladder. Capillary and arteriovenous hemangiomas are rare [7, 8]. The endoscopic findings of a sessile, blue, raised mass in a young patient who presents with macroscopic hematuria are highly suggestive of hemangioma. The endoscopic differential diagnostic considerations for pigmented raised lesions include endometriosis, melanoma, and sarcoma. Accurate diagnosis requires confirmation by biopsy. Histologically, CHB has the same characteristics of usual hemangioma except for its dilated, big, and full blood vessels covered by flat endothelium. It is distinguished from angiosarcoma and Kaposi sarcoma by its lack of cytologic atypia. Exuberant vascular proliferation may be observed in papillary polypoid cystitis and granulation tissue; however, these lesions contain prominent inflammation, which usually is not observed in hemangioma. The pressure of CHB may cause a destructive effect on its close tissues. Thus, in most cases surgical removal is needed. It rarely becomes malignant. Thrombocytopenic purpura, in addition to hemorrhage, is one of the major complications of CHB, which occurs most often in infancy and secondary to the rapid enlargement of the hemangioma [9]. Imaging studies, such as ultrasonography, pelvic arteriography, computed tomography scan, and magnetic resonance imaging, are helpful in defining the extent and location of the tumor. For small lesions, surveillance is sufficient. The treatment is only necessary when the lesions threaten the organ function or the patient′s performans status. The management of patients with hemangioma is controversial. Numerous therapeutic approaches are available, including trans-urethral resection and electrocoagulation, partial or complete cystectomy, sclerosing agent injection, irradiation, systemic steroids, and interferon-a-2 therapy, and, more recently, YAG-laser therapy [10]. Partial cystectomy is the treatment of choice for large and accessible lesions. It is limited by the diffuse lesions which are found in the bladder floor. Regarding to transurethral resection, the risk of uncontrollable bleeding is insignificant when the lesion is small (≤3 cm). Monopolar or bipolar electrocoagulation is effective [11]. Neodymium YAG laser becomes more and more as a first-line therapy. It allows a complete coagulation of the whole bladder thickness [12]. Selective arterial embolization has been used successfully for twice hemangiomas bladder and urethra [13]. Radiotherapy is indicated in non-surgical lesions. Some successes have been achieved, but also failures and recurrences [14].

## Conclusion

Cavernous hemangioma of bladder is rare. Electrocoagulation or YAG Laser is effective the lesion is small. Partial cystectomy is the standard treatment of voluminous and isolated lesions of the movable portion of the bladder especially in cases of local invasion Although CHB have a benign course and favorable outcome, follow up is mandatory to detect recurrence or residual disease.
